# Secretaries and Their Limitations

**Published:** 1889-12-07

**Authors:** 


### The Hospital

A Weekly Journal of

Science, Medicine, Nursing, and Philanthropy.

*For Hospitals, Asylums, Medical Practitioners, Students, Nurses, Families, Parishes, Congregations, and the Charitable Public.*

Vol. VII. No. 167.] [December 7, 1889.

### Secretaries and their Limitations.

The scandal at the City-road Chest Hospital has at last come to an end. So far as the initiated are concerned the matter might well end there. It would be difficult to find a single well-instructed hospital manager or right-minded secretary who would not agree with the decision that has been arrived at. But for the sake of that large public who, whilst not professing to understand much about hospital management, yet take a deep interest in the care and treatment of the sick poor, it will be well to point out certain important principles which have led the governors to a just and wise decision.

The facts of the case may be recapitulated in a few words. The late secretary of the City-road Hospital charged against members of the medical staff that they were inclined to make the hospital rather a school for medical instruction than for the benefit of the sick poor, and that they had performed certain operations on patients which were more of the nature of clinical experiments than of tried and proved methods of treatment. A careful and impartial enquiry elicited the undoubted certainty that those charges were not justified by actual facts. It was therefore considered advisable for the sake of avoiding friction between the medical staff and the secretary that the latter should resign, and he resigned forthwith. But this decision of the council does not appear to have been satisfactory to the secretary and certain supporters of the hospital who sympathised with him, and it was sought at a subsequent meeting to have it reversed. Thus matters stood until Thursday, November 28th, when a special court of governors was held at the Cannon-street Hotel for the purpose of dealing authoritatively and finally with the whole question. There was a crowded attendance, among other governors present being the Earl of Derby, Alderman Sir James Whitehead, Bart., Lord Charles Bruce, president of the hospital, Mr. S. Hope Morley, president of the council, Colonel Makins, and other well-known persons. The result of the renewed debate was the affirmation of the original decisions of the council, that the confidence of the managers in the medical staff was unabated, and that the resignation of the secretary be accepted as final.

We venture to express the emphatic opinion that this scandalous breach of the peace of the hospital was, both in its inception and in its continued progress, absolutely and entirely without justification. It arose from the one fatal mistake of the secretary in taking upon himself functions which did not by the constitution of the hospital appertain to him, and which he was in no sense fitted to perform. He constituted himself the critic and censor of the medical staff, and that on questions of which he could have no knowledge whatever. He assumed that he knew better than they, not only what patients should be admitted and how long they should remain in the hospital, but also what were and what were not proper methods of treatment to be applied in critical cases.

But what is the significance of assumptions like these? They mean that not only is the secretary the hand of the committee in the administration of the financial affairs of the hospital, but that he is the head both of the committee and of the medical staff in conducting the medical and surgical practice of the institution. But the secretary is not a qualified medical man; he is not even a medical student; nay, he has not received so much training as a matron or a nurse. Whence then comes his knowledge of the class of cases to be admitted, and still more of the methods of treatment to be practised? He has no knowledge at all. He is no more competent to decide either of these questions than is the hall porter or the hospital milkman. The ready proof of this lies in the fact that no secretary who understands his duties ever thinks of constituting himself the critic and adviser of the medical staff. The present case is the first of its kind that has ever occurred within the writer's twenty years' experience of hospital life and management.

The governors of the Chest Hospital have deserved well of the public in that they have taken a comprehensive and rational view of the situation. No doubt it is open to hypercritical persons to ask if the medical staff of any and every hospital is to be given an absolutely free hand both as to the admission and the treatment of patients; and is to be subjected to no external supervision or criticism of any kind. The asking of the question gives us an opportunity of explaining exactly how affairs are regulated in this regard. As a matter of fact, the committee of any given hospital usually consists of educated laymen, with a sprinkling of medical men. It is the final board of appeal, subject to the control of the governors, in all cases of internal management. Next in order of importance is the house committee. These two bodies supervise the work and conduct of the medical staff, so far as such supervision is required, regularly and punctually. There is, therefore, no question whatever about the medical staff having a perfectly free hand to do what they please. They have not a free hand. Like every other department of administration, the work of the medical staff is under the watchful care of a competent committee of management.

But supposing that the staff of every hospital throughout Great Britain were not subject to the control of a committee, and had a perfectly free hand to do exactly what they pleased, what would happen? This, that every patient in every hospital in the kingdom would be treated exactly as he is at the present time. If it is asked, What are the checks upon the abuse of medical liberty in hospitals? the answer is that the checks are innumerable, and absolutely efficient. First and chiefly is the check found in every hospital physician's own heart—a heart that impels him with irresistible impulsion to do to the patient as he would wish to be done by. That of itself is amply sufficient to ensure the best and kindest treatment to every inmate of a hospital. Then is there not the constant check of the watchful eye of the professional brother who is also on the staff, and who is not seldom the professional rival? But more than that is the *esprit de corps* of the whole staff, which makes it critically jealous of the reputation of the institution, and of the competency or otherwise of each medical officer in whose hands that reputation is placed. These and the like checks are in constant operation, and that they are amply sufficient is proved by the fact that not once in ten years is any hospital staff called to account for misconduct or mistake.

The public may rest assured that what has been done by the governors of the Royal Chest Hospital has been both just and wise. The secretary, possibly from excess of well-intentioned zeal, made a grave mistake, and he has suffered for it. That is a misfortune for him undoubtedly; but men have to find out that mistakes are punished in this world, as well as wilful faults. But though the secretary has suffered, it is much to be hoped that the hospital itself will not suffer. The recent investigations have proved that it is admirably manned, from a medical point of view, and excellently fitted to do the work which it was founded to do. They have also shown to demonstration that its general affairs are in the hands of competent and trustworthy persons, who—at critical as well as ordinary times—are equal to the duties imposed upon them. We therefore venture to bespeak for the institution, at this important juncture, a more than ordinary degree of consideration, and a larger measure of sympathetic help.

